# Prevalence and clinical correlates of Peyronie’s disease in patients with Dupuytren’s disease: a cross-sectional study from a tertiary andrology center

**DOI:** 10.1093/sexmed/qfaf083

**Published:** 2025-10-14

**Authors:** Gökhan Çeker, Ertuğrul Arıkız, Akif Erbin, Hakan Anıl, Halil Lütfi Canat

**Affiliations:** Department of Urology, Basaksehir Cam and Sakura City Hospital, Istanbul, 34480, Turkey; Department of Histology and Embryology, Hamidiye Institute of Health Sciences, University of Health Sciences, Istanbul, 34668, Turkey; Department of Urology, Basaksehir Cam and Sakura City Hospital, Istanbul, 34480, Turkey; Department of Urology, Basaksehir Cam and Sakura City Hospital, Istanbul, 34480, Turkey; Department of Urology, Adana City Training and Research Hospital, Adana, 01230, Turkey; Department of Urology, Basaksehir Cam and Sakura City Hospital, Istanbul, 34480, Turkey

**Keywords:** diabetes, Dupuytren's contracture, Dupuytren's disease, palmar fibromatosis, penile fibromatosis, Peyronie's disease

## Abstract

**Background:**

Dupuytren’s disease (DD) and Peyronie’s disease (PD) are fibroproliferative disorders that may share common pathophysiological mechanisms.

**Aim:**

To determine the prevalence of PD among male patients diagnosed with DD and to investigate its clinical and laboratory correlates.

**Methods:**

This cross-sectional observational study was conducted at a tertiary academic center and included 101 male patients diagnosed with DD. All participants underwent structured andrological evaluation and were classified into PD and non-PD groups. Clinical and laboratory parameters—including demographic, metabolic, sexual function, and inflammatory markers—were compared between the groups. Statistical analyses included descriptive statistics, Chi-square or Fisher’s exact tests for categorical variables, and Mann–Whitney U tests for continuous variables. Binary logistic regression was performed to identify independent predictors of concomitant PD.

**Outcomes:**

The primary outcome was the prevalence of PD among patients with DD. Secondary outcomes included associations between PD and clinical/laboratory features.

**Results:**

PD was significantly associated with bilateral Dupuytren’s contracture (86.4% vs. 50.6%, *P* = 0.003), diabetes mellitus (81.8% vs. 44.3%, *P* = 0.003), lower high-density lipoprotein cholesterol (median 38 vs. 43 mg/dL, *P* = 0.030), higher fasting glucose (median 146 vs. 104 mg/dL, *P* = 0.018), and higher HbA1c (median 7.55% vs. 6.20%, *P* = 0.005).

Erectile function, assessed by the Erection Hardness Score, differed significantly between groups (median 3 in both; interquartile range: 2–3 in the PD group vs. 3–4 in the non-PD group, *P* = 0.007) (no significant differences were observed in the 6-item International Index of Erectile Function and the Sexual Health Inventory for Men scores).

In multivariate analysis, bilateral DD (OR = 17.80, *P* = 0.020) and HbA1c (OR = 1.589, *P* = 0.031) remained independently associated with PD. Other variables did not differ significantly between groups.

**Clinical Implications:**

Male patients with DD—especially those with bilateral hand involvement or poor glycemic control—may warrant consideration for opportunistic urological evaluation to identify concomitant PD. However, further research is needed to determine the impact of early detection on patient outcomes.

**Strengths and Limitations:**

This study is among the few to explore the prevalence and clinical correlates of PD in patients with DD, using a structured urological evaluation and comprehensive laboratory profiling. However, its cross-sectional design limits causal inference, and the single-center setting may affect the generalizability of the findings.

**Conclusion:**

PD is highly prevalent among patients with DD, especially those with bilateral contracture and poor glycemic control. These findings support a possible systemic fibrotic predisposition and highlight the value of integrated metabolic and sexual health assessment in this population.

## Introduction

Peyronie’s disease (PD) is a progressive fibrotic disorder of the tunica albuginea of the penis, characterized by localized plaque formation, penile deformity, and, in many cases, pain or erectile dysfunction. Although the exact pathophysiology of PD remains incompletely understood, it is widely accepted that repetitive microvascular trauma to the tunica albuginea during sexual activity may trigger aberrant wound healing and fibrotic changes mediated by proinflammatory and profibrotic cytokines, particularly transforming growth factor-beta 1 (TGF-β1).^1^^,^^2^ In addition, previous case–control research by Bjekic et al. has identified several systemic and lifestyle-related comorbidities, including diabetes mellitus, hypertension, smoking, and a history of Dupuytren’s contracture, as independent predictors of PD, supporting the concept of shared pathogenic pathways between these conditions and fibromatous disorders.^3^

A potential association between PD and systemic fibrotic diatheses has long been hypothesized. Dupuytren’s disease (DD), a fibroproliferative condition of the palmar fascia, shares common histopathological features with PD, including increased myofibroblast activity, excessive collagen deposition, and a similar cytokine milieu.^4^^,^^5^ Several epidemiological studies have reported a significantly higher prevalence of PD in patients with DD, suggesting a shared pathogenic mechanism or genetic predisposition to abnormal fibrotic tissue remodeling.^6^^,^^7^

Despite the proposed link, the extent of this association remains unclear. In particular, little is known about which clinical or laboratory features might distinguish DD patients with concomitant PD from those without. While prior research has largely focused on self-reported symptoms or survey-based data, few studies have incorporated structured urological assessment alongside metabolic and laboratory profiling. This limits the identification of clinically useful predictors or screening strategies for PD in DD populations.

Given that both disorders may reflect a systemic fibrotic predisposition, investigating potential associations with metabolic comorbidities—such as diabetes mellitus and dyslipidemia—and objective markers of glycemic control like glycated hemoglobin (HbA1c) is of particular clinical interest. Additionally, while sexual dysfunction is a well-documented feature of PD, its evaluation has rarely been integrated into DD-focused studies, despite plausible overlap.

Accordingly, this study aimed to determine the prevalence of PD in a cohort of male patients with DD using standardized genital examination. It also sought to explore clinical and laboratory correlates of concomitant PD, including metabolic parameters, comorbidities, and erectile function scores. A better understanding of these associations may facilitate earlier recognition and comprehensive management of men affected by both conditions. Specifically, this study investigates: In men with DD, what is the prevalence of PD, and which clinical or metabolic factors are associated with its presence?

## Methods

### Study design and setting

**Figure 1:**
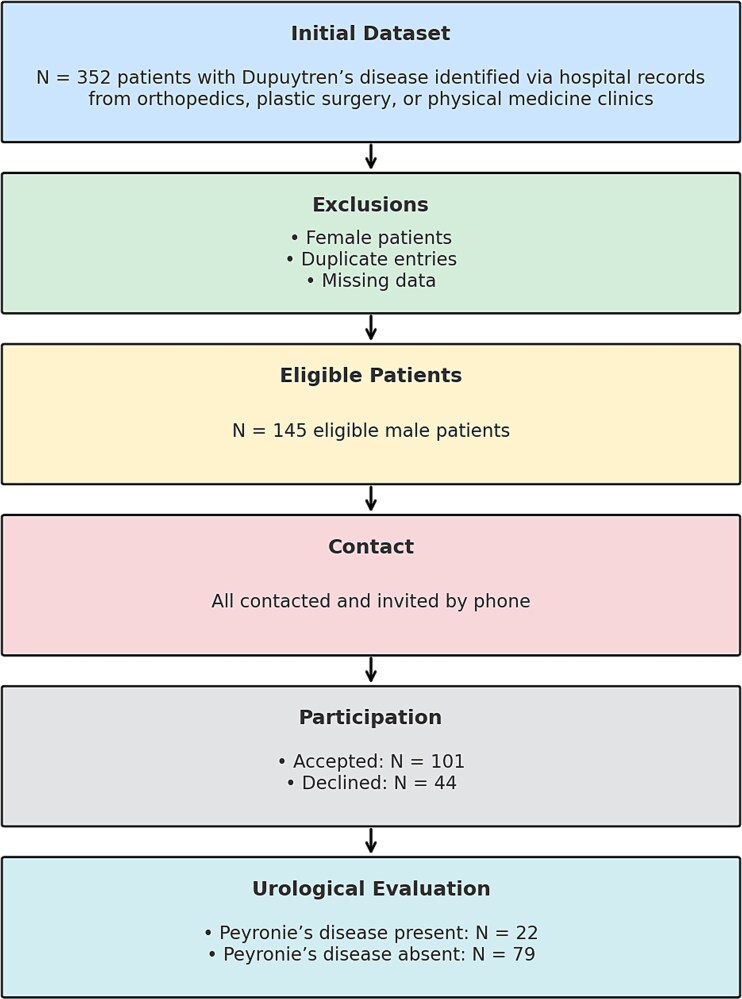
Study population and analysis flowchart.

This cross-sectional observational study was conducted at Başakşehir Çam and Sakura City Hospital, a tertiary academic medical center, between 15 August 2024 and 15 May 2025. A total of 352 patient entries with a diagnosis of Dupuytren’s contracture were initially identified in the hospital database, across orthopedic, plastic surgery, and physical medicine outpatient clinics. Following data cleaning procedures, duplicate entries, female patients, and those with incomplete clinical records were excluded. After exclusions, 145 unique male patients aged ≥18 years were contacted by phone. During the call, patients were informed that DD may be associated with urological conditions and were invited to attend a detailed urological evaluation as part of the study. A total of 101 patients consented to participate and attended the urology outpatient clinic for inclusion in the final study cohort. These 101 patients were subsequently categorized into two groups—22 with concomitant PD and 79 without PD—and were comparatively analyzed for clinical and laboratory predictors (Figure 1).

The primary aim of this study was to determine the prevalence of PD among men diagnosed with DD. Secondary analyses focused on identifying clinical and laboratory correlates of concomitant PD. Relevant demographic, clinical, and laboratory parameters are summarized in the subsequent section.

### Clinical and laboratory assessment

To ensure diagnostic consistency, every patient was jointly assessed by two andrology specialists, each with over five years of clinical experience in male sexual health. The diagnosis of PD was established through standardized physical examination, based on the presence of palpable fibrous plaques, penile curvature, and/or symptoms such as pain during erection.^5^ No imaging modalities were used; diagnosis relied exclusively on clinical findings. Consistent with the 2025 EAU Guidelines on Sexual and Reproductive Health,^8^ the diagnosis of PD in this study relied exclusively on clinical history and physical examination; routine imaging modalities (eg, ultrasound, CT, MRI) were not performed, except in select cases as recommended by the guidelines. The degree and direction of penile curvature were assessed by visual inspection during erection, primarily based on patient self-report and home photographs taken in the erect state. In patients with severe erectile dysfunction who were unable to achieve sufficient rigidity at home, an office-based pharmacologically induced erection was performed to allow accurate assessment of curvature. For all patients diagnosed with PD, detailed phenotypic characteristics—including plaque size, location, and degree/direction of penile curvature—were systematically recorded.

Demographic data including age, height, weight, and body mass index (BMI); laterality of Dupuytren’s contracture (unilateral or bilateral); and a comprehensive set of comorbidities and relevant medical history—such as diabetes mellitus, hypertension, hyperlipidemia, cataract, Ledderhose disease, Parkinson’s disease, epilepsy, rheumatoid arthritis, and beta-blocker use—were recorded. In addition, smoking history, alcohol consumption, family history of PD and/or DD, and previous penile trauma or fracture were documented.

Sexual function was assessed using the Sexual Health Inventory for Men (SHIM) and the 6-item International Index of Erectile Function (IIEF-6). Erection quality was further evaluated using the Erection Hardness Score (EHS). As part of the structured andrological evaluation, all patients were specifically questioned regarding the presence of penile curvature and pain during erection, as well as other sexual complaints including penile pain during intercourse, partner-related pain, difficulty with vaginal penetration, and impaired coital movements. However, standardized disease-specific questionnaires, such as the Peyronie’s Disease Questionnaire (PDQ), were not routinely applied in this cohort.

Laboratory parameters were retrieved from hospital electronic medical records and included:

- ***Complete blood count**:* hemoglobin, neutrophils, lymphocytes, eosinophils, monocytes, and platelets.
- ***Inflammatory ratios**:* neutrophil-to-lymphocyte ratio (NLR), eosinophil-to-lymphocyte ratio (ELR), monocyte-to-eosinophil ratio, neutrophil-to-eosinophil ratio, and platelet-to-lymphocyte ratio.
- ***Metabolic and hormonal profile:*** fasting plasma glucose, glycated hemoglobin (HbA1c), total testosterone, triglycerides (TG), total cholesterol, high-density lipoprotein (HDL), low-density lipoprotein (LDL), and TG/HDL ratio, a surrogate marker of insulin resistance (IR).

All clinical and laboratory data were anonymized and systematically recorded using a standardized case report form.

### Statistical analysis

All statistical analyses were performed using IBM SPSS Statistics for Windows, version 27.0 (IBM Corp., Armonk, NY, USA). The normality of continuous variables was assessed using the Shapiro–Wilk test. As most continuous variables deviated from normal distribution, non-parametric tests were applied. The Mann–Whitney U test was used to compare continuous variables between patients with and without PD, and Chi-square or Fisher’s exact test was used for categorical variables, as appropriate. Correlations between variables were evaluated using Spearman’s rank correlation coefficient.

A multivariate analysis was conducted using forward stepwise binary logistic regression (likelihood ratio method) to identify independent predictors of concomitant PD. Variables were included based on clinical relevance or univariate significance. Odds ratios (ORs) with 95% confidence intervals (CIs) were calculated. Additionally, receiver operating characteristic (ROC) curve analysis was performed for the continuous variable that remained significant in the multivariate model to assess its discriminative ability. The area under the curve (AUC), 95% CI, sensitivity, specificity, and optimal cut-off value (based on the maximum Youden index) were reported. A two-tailed *P*-value < 0.05 was considered statistically significant.

### Ethical approval

Ethical approval for the study was obtained from the Ethics Committee of Başakşehir Çam and Sakura City Hospital (Approval No: KAEK-11/24.07.2024.80). All procedures were conducted in accordance with the principles of the Declaration of Helsinki.

## Results

A total of 101 male patients diagnosed with DD were included in the final analysis. Among these, 22 patients (21.8%) were found to have concomitant PD. The groups were similar in terms of basic demographic data. Bilateral Dupuytren’s contracture was significantly more frequent among patients with PD than those without (86.4% vs. 50.6%, *P* = 0.003). Similarly, the prevalence of diabetes mellitus was significantly higher in the PD group (81.8% vs. 44.3%, *P* = 0.003) (Table 1).

**Table 1:** Comparison of clinical parameters between PD and non-PD groups.

	**PD group (n = 22)**	**Non-PD group (n = 79)**	***P* value**
Current age (y)	57 (52-65)	60 (48-69)	0.319
BMI	27.4 (23.9-32.4)	27 (23.8-31.7)	0.524
Cigarette smoking, n (%)			0.530
Current smoker Former smoker Never smoked	4 (18.2%) 7 (31.8%) 11(50.0%)	21 (26.6%) 17 (21.5%) 41 (51.9%)	
Alcohol use, n (%)	1 (4.5%)	3 (3.8%)	0.874
Laterality of DD, n (%)			0.003
Unilateral Bilateral	3 (13.6%) 19 (86.4%)	39 (49.4%) 40 (50.6%)	
History of trauma (n, %)	1 (4.5%)	0 (0.0%)	0.218
History of penile fracture (n, %)	1 (4.5%)	0 (0.0%)	0.218
Diabetes mellitus, n (%)	18 (81.8%)	35 (44.3%)	0.003
Hypertension, n (%)	12 (54.5%)	35 (44.3%)	0.394
CAD, n (%)	2 (9.1%)	1 (1.3%)	0.119
Hyperlipidemia, n (%)	5 (22.7%)	21 (26.6%)	0.715
Ledderhose disease, n (%)	1 (4.5%)	2 (2.5%)	0.525
Rheumatoid arthritis, n (%)	1 (4.5%)	4 (5.1%)	0.921
Cataract, n (%)	2 (9.1%)	16 (20.3)	0.347
Glaucoma, n (%)	0 (0.0%)	4 (5.1%)	0.574
Topical Timolol use, n (%)	0 (0.0%)	3 (3.8%)	0.353
Gout, n (%)	0 (0.0%)	2 (2.5%)	0.451
Epilepsy, n (%)	0 (0.0%)	1 (1.3%)	0.596
Multiple Sclerosis, n (%)	0 (0.0%)	3 (3.8%)	0.353
Parkinson’s Disease, n (%)	0 (0.0%)	2 (2.5%)	0.451
Family History of DD (n, %)			0.293
1st-degree relative 2nd-degree relative	1 (4.5%) 0 (0.0%)	12 (15.2%) 1 (1.3%)	
Family History of PD (n, %)	0 (0.0%)	0 (0.0%)	

Categorical variables are presented as number (percentage); continuous variables are presented as median (25th–75th percentile).

PD group: for patients with Peyronie’s disease

Non-PD group: for patients without Peyronie’s disease

PD: Peyronie’s disease

DD: Dupuytren’s disease

CAD: Coronary artery disease

Erectile function parameters are summarized in Table 2. While SHIM and IIEF-6 scores tended to be lower in the PD group, only the EHS differed significantly between groups (*P* = 0.007); although the median value was the same (3), the interquartile range was narrower in the non-PD group, reflecting better erectile rigidity.

**Table 2:** Sexual function characteristics in patients with and without PD.

	**PD group (n = 22)**	**Non-PD group (n = 79)**	***P* value**
Erectile dysfunction, n (%)	20 (90.9%)	55 (69.6%)	0.054
SHIM	16 (1-21)	20 (9-24)	0.052
IIEF-6	20 (14-28)	23 (12-29)	0.093
EHS	3 (2-4)	3 (3-4)	0.007
Pain in erection or coitus, n (%)	1 (4.5%)	2 (2.5%)	0.525
Partner pain during coitus, n (%)	0 (0.0%)	1 (1.3%)	0.596
Intromission difficulty, n (%)	1 (4.5%)	4 (5.1%)	0.921
Coital movement difficulty, n (%)	0 (0.0%)	2 (2.5%)	0.451

Categorical variables are presented as number (percentage); continuous variables are presented as median (25th–75th percentile).

SHIM: Sexual Health Inventory for Men

IIEF-6: International Index of Erectile Function – 6-item version

EHS: Erection Hardness Score

Exploratory correlation analysis revealed a strong positive association between EHS and both SHIM (ρ = 0.906, *P* < 0.001) and IIEF-6 scores (ρ = 0.891, *P* < 0.001). Additionally, men with EHS ≤ 2 had significantly lower SHIM scores (median [IQR]: 9 [1–15] vs. 21 [18–24]) and IIEF-6 scores (median [IQR]: 12 [1–16] vs. 25 [22–28]) compared to those with EHS ≥ 3 (both *P* < 0.001).

Among laboratory parameters, only fasting plasma glucose (median 146 mg/dL vs. 104 mg/dL, *P* = 0.018), HbA1c (median 7.55% vs. 6.20%, *P* = 0.005), and HDL cholesterol (median 38 mg/dL vs. 43 mg/dL, *P* = 0.030) showed statistically significant differences between groups. Other biochemical and inflammatory markers, including triglyceride-to-HDL cholesterol ratio (a surrogate marker of IR) and neutrophil-to- ratio, were similar between groups (Table 3).

**Table 3:** Laboratory parameters and biomarkers in patients with and without PD.

	**PD group (n = 22)**	**Non-PD group (n = 79)**	***P* value**
Hemoglobin (g/dL)	14.6 (12.8-15.9)	14.5 (13.1-15.9)	0.834
Neutrophil count (×10^9^/L)	5.05 (3.28-5.87)	4.48 (3.47-6.25)	0.397
Lymphocyte count (×10^9^/L)	2.36 (1.20-3.40)	2.23 (1.40-2.83)	0.202
Eosinophil count (×10^9^/L)	0.19 (0.04-1.25)	0.15 (0.09-0.30)	0.624
Monocyte count (×10^9^/L)	0.62 (0.50-0.93)	0.61 (0.46-0.76)	0.492
Platelet count (×10^9^/L)	233.5 (188-315)	232 (192-293)	0.977
Neutrophil/Lymphocyte	2.1 (1.3-4.1)	2.1 (1.4-3.8)	0.928
Neutrophil/Eosinophil	26.9 (12.6-110.0)	30.0 (14.1-60.8)	0.977
Monocyte/Eosinophil	3.29 (1.58-11.00)	3.81 (2.03-6.60)	0.892
Platelet/Lymphocyte	100.2 (76.8-160.0)	106.7 (80.8-154.8)	0.422
Total Cholesterol (mg/dL)	139 (113-196)	157 (120-196)	0.379
LDL Cholesterol (mg/dL)	107 (71-152)	106 (79-145)	0.954
HDL Cholesterol (mg/dL)	38 (31-59)	43 (36-60)	0.030
Triglycerides (TG) (mg/dL)	124.5 (78.0-283.0)	120.5 (73.0-203.0)	0.417
Insulin resistance (IR) (TG/HDL-C) ratio	3.55 (1.87-6.15)	2.86 (1.40-5.58)	0.108
Fasting plasma glucose (mg/dL)	146 (93-202)	104 (86-190)	0.018
Glycated hemoglobin (HbA1c, %)	7.55 (6.70-15.40)	6.20 (5.40-9.40)	0.005
Total testosterone (ng/mL)	3.84 (2.55-5.62)	4.26 (2.96-5.97)	0.221

Continuous variables are presented as median (25th–75th percentile).

The phenotypic features of patients diagnosed with PD—including plaque size, location, and curvature degree/direction—are presented in Table 4.

**Table 4:** Detailed phenotypic characteristics of patients with PD in the study cohort.

**Patient number**	**Plaque location**	**Plaque size (mm)**	**Direction of curvature**	**Degree of curvature (°)**
**1**	Lateral (Right)	5	Right	30
**2**	Lateral (Left)	5	Left	30
**3**	Lateral (Left)	5	Left	45
**4**	Midshaft circumferential	10	Hourglass deformity	NA^*^
**5**	Dorsal	5	Upward	20
**6**	Dorsolateral (Right)	6	Dorsolateral right	30
**7**	Lateral (Left)	5	Left	20
**8**	Dorsal	5	Upward	20
**9**	Lateral (Right)	5	Right	45
**10**	Dorsolateral (Right)	4	Dorsolateral right	30
**11**	Lateral (Left)	4	Left	20
**12**	Dorsolateral (Right)	18	Dorsolateral right	30
**13**	Dorsal	5	Upward	40
**14**	Lateral (Left)	25	Left	30
**15**	Dorsal	30	Upward	50
**16**	Lateral (Right)	10	Right	40
**17**	Lateral (Right)	6	Right	30
**18**	Ventrolateral (Left)	10	Ventrolateral left	30
**19**	Lateral (Left)	4	Left	30
**20**	Lateral (Left)	5	Left	20
**21**	Lateral (Left)	4	Left	30
**22**	Ventral	5	Downward	20

^*^NA: Not applicable

A stepwise binary logistic regression analysis was performed to identify independent predictors of concomitant PD. Candidate variables entered into the model included diabetes mellitus, bilateral DD, HDL cholesterol, fasting glucose, HbA1c, insulin resistance (TG/HDL ratio), and EHS (Table 5). In the final multivariate model, two factors remained independently associated with PD:

- Bilateral Dupuytren’s contracture (OR = 17.80, 95% CI: 1.62–20.10, *P* = 0.020)
- HbA1c level (OR = 1.589, 95% CI: 1.04–2.42, *P* = 0.031)

**Table 5:** Stepwise logistic regression identifying independent risk factors for PD in patients with Dupuytren’s contracture.

	Univariate			Multivariate		
Variables	Odds ratio	P value	95% CI	Odds ratio	P value	95% CI
Diabetes mellitus	(No, reference)					
Yes	5.657	0.004	1.75–18.24			
IR	1.219	0.291	0.97–1.38			
EHS	0.657	0.029	0.45–0.95			
Dupuytren contracture	(Unilateral, reference)					
Bilateral	6.175	0.006	1.69–22.54	17.80	0.020	1.62–20.10
HDL, mg/dL	0.950	0.063	0.90–1.00			
Glucose, mg/dL	1.002	0.412	0.99–1.01			
HbA1c	1.438	0.023	1.05–1.96	1.589	0.031	1.04–2.42

IR: TG/HDL ratio, a surrogate marker of insulin resistance

EHS: Erection Hardness Score

HDL: High-density lipoprotein

HbA1c: Glycated hemoglobin

**Figure 2:**
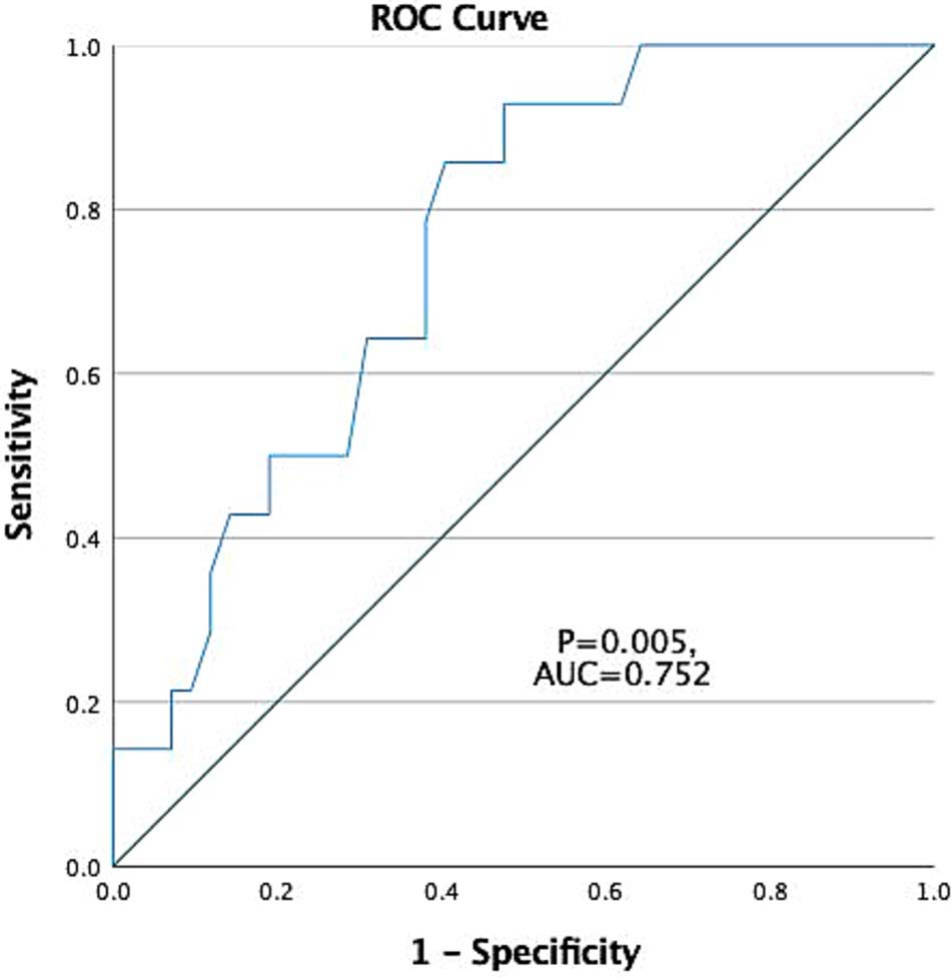
ROC curve illustrating the discriminatory performance of HbA1c for PD. The AUC was 0.752 (95% CI [0.620–0.883]), with a cut-off value of 6.65 yielding 85.7% sensitivity and 60.5% specificity. The diagonal line represents a random classifier (AUC = 0.5).

Additionally, a ROC curve analysis was performed to evaluate the discriminatory ability of HbA1c in identifying patients with concomitant PD. The area under the ROC curve (AUC) was 0.752 (95% CI: 0.620–0.883, *P* = 0.005), indicating a good predictive value. The optimal cut-off value of HbA1c was determined as 6.65%, yielding a sensitivity of 85.7% and a specificity of 60.5%, based on the maximum Youden index (Figure 2).

### Clinical interventions and management

Among patients diagnosed with PD, two were started on penile traction device therapy specifically for PD. In two additional patients, surgical intervention for PD was planned after achieving better glycemic control. All diabetic patients were referred to endocrinology for management of metabolic status. Oral phosphodiesterase type 5 inhibitors were initiated in five patients with clinically significant erectile dysfunction, and one patient was started on testosterone replacement therapy for documented hypogonadism.

## Discussion

The intersection between PD and DD represents an underexplored domain within the broader category of fibrotic disorders, despite longstanding hypotheses suggesting a shared pathophysiological origin.^6^^,^^7^^,^^9^ Both conditions are characterized by localized fibroproliferative changes, driven by aberrant myofibroblast activity, excessive collagen deposition, and dysregulated wound healing.^9^ While prior reports have largely examined the prevalence of DD among patients already diagnosed with PD,^6^ or assessed PD-like symptoms through self-reported questionnaires in DD cohorts,^10^ such approaches inherently limit diagnostic accuracy due to the absence of physical examination and objective confirmation. Within this context, the present study offers novel and clinically relevant insights as one of the first to implement a structured urological assessment, including detailed genital examinations by experienced andrologists, in a well-characterized DD cohort. This design enables a more reliable determination of PD prevalence and allows for investigation of its independent clinical and metabolic correlates.

The advantages of employing a structured clinical assessment become more apparent when compared to previous investigations. For instance, Mohede et al. reported an 8.8% self-reported PD rate in 730 DD patients,^7^ whereas Shindel et al. identified Peyronie’s-like symptoms in 26% of surveyed DD patients using a self-administered questionnaire.^10^ However, in both studies, objective confirmation of PD through physical examination was not performed, possibly leading to misclassification. In our study, diagnosis was based on clinical confirmation by two independent andrologists, a method by which subjectivity was minimized and diagnostic specificity enhanced.

A concomitant diagnosis of PD was identified in 21.8% of patients with DD, a rate considerably higher than that reported in the general male population (3-5%).^11^^,^^12^ This observation lends further support to the hypothesis of a systemic fibrotic diathesis, in which fibroproliferative processes may extend beyond localized anatomical regions. Notably, bilateral Dupuytren’s contracture was found to be a strong independent predictor of PD, with an odds ratio of 17.80. Importantly, this study is the first to identify bilateral Dupuytren’s involvement as an independent predictor of PD, suggesting that more extensive palmar involvement may reflect a greater systemic fibrotic predisposition. While previous studies, such as Nugteren et al., have established the coexistence of DD and PD, the role of bilateral DD has not been previously explored or quantified.^6^

Beyond clinical and metabolic correlates, genetic predisposition represents another important dimension in the shared pathophysiology of DD and PD. An accumulating body of evidence supports a strong genetic predisposition underlying both DD and PD, reinforcing the hypothesis of a shared fibroproliferative diathesis. Family history of DD confers a substantially increased risk, with prior studies indicating a 2.9- to 4.5-fold higher prevalence among siblings of affected individuals, and heritability estimates reaching up to 80% based on large-scale twin studies.^13–15^ Similarly, PD has been reported to follow an autosomal dominant inheritance pattern with incomplete penetrance, particularly in families where both DD and PD coexist. Qian et al. demonstrated overlapping gene expression profiles in affected tissues of PD and DD patients, notably in genes regulating myofibroblast differentiation, collagen metabolism, and oxidative stress pathways.^4^ In the present cohort, a positive family history of DD was documented in 4.5% (1/22) of individuals with concomitant PD and DD, compared to 15.2% (12/79) of those with isolated DD. No patients in either group reported a family history of PD. While the heritability of DD is well established, further studies with larger sample sizes and genetic analyses are warranted to elucidate the role of inherited factors in the development of PD among individuals with DD. In this regard, the population-based study by Allen-Brady et al. provides compelling evidence of significant familial clustering of PD extending to distant relatives, suggesting that heritable factors may underlie at least a subset of PD cases, particularly when coexisting with other fibromatous disorders such as DD.^16^

Another key finding is the independent association between elevated HbA1c levels and the presence of PD. Chronic hyperglycemia has been implicated in fibrotic tissue remodeling through several mechanisms, including upregulation of TGF-β1, accumulation of advanced glycation end-products, and oxidative stress.^17^ Our results align with this pathophysiological framework and suggest that metabolic dysregulation may contribute to the development or progression of PD in patients with DD.^9^ Notably, this association persisted even after controlling for diabetes mellitus status in the multivariate model, indicating that subclinical glycemic derangements may still play a role. In line with this, El-Sakka and Tayeb, in a large cohort of men with type 2 diabetes screened for erectile dysfunction, reported a PD prevalence of 8.1% and found that longer diabetes duration and poor metabolic control (higher HbA1c) were associated with greater PD risk.^18^ Similarly, Pavone et al. reported that smoking and hypertension were significant PD risk factors, although they did not find a significant association with diabetes, highlighting possible population-specific differences.^19^

Interestingly, although low HDL cholesterol and elevated fasting glucose were associated with PD in univariate analysis, these variables did not retain significance in the multivariate model. Similarly, the triglyceride-to-HDL ratio—an indirect marker of insulin resistance— demonstrated a non-significant association, although a trend was observed. These results suggest that HbA1c may serve as a more stable and integrative biomarker of chronic metabolic burden in the context of fibrotic risk than single time-point lipid or glucose measures.^20^

Notably, inflammatory markers—including NLR and ELR—did not significantly differ between patients with and without PD. Although such markers have been shown to aid in the differentiation between acute and chronic phases of fibrotic diseases like PD,^21^^,^^22^ their relevance appears limited in cohorts where both DD and PD have progressed to a stable, chronic phase. These findings suggest that, once the fibrotic process has matured, systemic inflammatory markers may no longer be reflective of disease activity. Further studies are warranted to determine their potential role in early or progressive stages of these conditions.

In the present study, erectile function assessed via the EHS was found to be significantly lower in men with PD, whereas SHIM and IIEF-6 scores did not differ meaningfully between groups. This discrepancy may be attributed to the greater sensitivity of the EHS in detecting rigidity impairments related to anatomical changes—such as fibrotic plaque formation or penile curvature—which are hallmark features of PD but may not be adequately captured by broader, multi-item erectile function instruments. Although the association between EHS and PD did not remain significant in the multivariable model, the univariate difference supports the notion that rigidity-specific assessments may offer added clinical value in patients with structural erectile dysfunction. Notably, when patients were stratified by rigidity level, those with EHS ≤ 2 also demonstrated substantially lower SHIM and IIEF-6 scores compared to patients with EHS ≥ 3, indicating that reduced penile rigidity is associated with broader impairments in sexual function. This finding suggests that EHS, while primarily a measure of mechanical rigidity, may also indirectly reflect the overall sexual impact of PD. The importance of incorporating both functional and rigidity-specific measures was underscored by previous studies, in which EHS was shown to correlate with IIEF-EF while focusing more directly on mechanical rigidity, a critical determinant of penetrative sexual activity.^23^^,^^24^

Although this study did not demonstrate a statistically significant association between PD and certain systemic or localized conditions—such as multiple sclerosis (MS), rheumatoid arthritis (RA), Parkinson’s disease, gout, epilepsy, cataract, glaucoma, or Ledderhose disease—it remains important to consider their potential relevance within the broader context of fibroproliferative disorders. DD, PD, and Ledderhose disease are often cited as manifestations of a shared fibrotic diathesis.^25^ While our findings did not confirm a significant overlap between PD and Ledderhose disease in our cohort, this association has been described in the literature and may suggest a variant expression of the same pathological mechanism involving dysregulated myofibroblast activity.^7^ Similarly, while topical beta-blocker use, particularly timolol, did not show a significant link to PD in our population, previous reports have identified cases of synchronous PD and DC following topical timolol administration, implicating possible systemic absorption and profibrotic effects of beta-adrenergic blockade.^26^^,^^27^

Neurological conditions such as epilepsy, MS, and Parkinson’s disease have also been investigated for their associations with DC or PD, albeit with inconsistent findings.^28^ For instance, knuckle pads, considered part of the Dupuytren diathesis spectrum, have been observed more frequently among epileptic patients, possibly due to anticonvulsant-induced connective tissue changes or repetitive mechanical trauma.^29^ Moreover, an older hypothesis suggested a link between DC and alcohol-related cataracts, likely as part of a broader systemic fibrotic or metabolic response to chronic alcohol exposure, though this has not been substantiated in large-scale studies.^30^ Despite these historical and anecdotal associations, the current understanding of PD pathogenesis prioritizes local microtrauma, genetic susceptibility, and systemic metabolic dysfunction (eg, insulin resistance, oxidative stress, or hypogonadism) as central contributors.^31^ Consequently, while the aforementioned conditions may not independently predict PD risk, they remain relevant in the context of overlapping systemic or connective tissue pathologies and warrant further investigation in larger, longitudinal cohorts.

From a clinical standpoint, our findings suggest that targeted urological evaluation may be considered in men with DD who have bilateral involvement or elevated HbA1c. However, the clinical utility of such screening remains to be confirmed by future prospective studies assessing patient-reported outcomes and therapeutic implications. Given that PD is often underreported due to embarrassment or misconceptions about its treatability or aging-related nature,^32^^,^^33^ opportunistic screening in high-risk populations such as men with DD may improve early diagnosis and outcomes. Early recognition also enables timely counseling about non-surgical treatment options, sexual function rehabilitation, and potential progression. While it is possible that the identification of subclinical (asymptomatic) PD could lead to patient anxiety or overtreatment in some settings, in our cohort, the detection of PD—including in patients not actively seeking sexual health care—enabled timely counseling and evidence-based management. Specifically, two patients received penile traction device therapy, two were scheduled for surgical intervention after glycemic optimization, all diabetic patients were referred to endocrinology, five patients with significant erectile dysfunction were started on phosphodiesterase type 5 inhibitors, and one patient was treated for hypogonadism. These targeted interventions underscore the potential clinical benefit of identifying PD in this population, rather than resulting in unnecessary or inappropriate treatments. Nevertheless, further studies are needed to clarify the long-term impact of such interventions, particularly in asymptomatic individuals.

Despite its novel findings, this study has several limitations that should be acknowledged. First, the cross-sectional design limits causal inference, as temporal relationships between metabolic or inflammatory parameters and PD onset cannot be determined. Second, the study was conducted in a single tertiary care center, which may introduce referral bias and limit the generalizability of findings to broader populations, particularly those not routinely evaluated by andrology specialists. Third, the sample size, while adequate for detecting statistically significant associations, remains modest—especially in the PD subgroup—and may limit the power to identify smaller effect sizes. Fourth, while sexual function was assessed using the SHIM, IIEF-6, and EHS instruments, data on psychological burden and patient-reported quality of life were not systematically collected, and standardized disease-specific questionnaires such as the PDQ were not utilized; this represents a limitation of our study. Lastly, the use of a single time-point laboratory assessment precludes evaluation of dynamic changes in inflammatory or metabolic markers that may better reflect disease activity or progression. Future prospective, multicenter studies with larger cohorts and longitudinal data are warranted to validate and expand upon these findings.

## Conclusions

This study demonstrates a markedly increased prevalence of PD among patients with DD, particularly in those with bilateral hand involvement and suboptimal glycemic control. The identification of bilateral Dupuytren’s contracture as an independent risk factor for PD underscores the likelihood of a shared systemic fibrotic diathesis. By employing structured physical examination by andrology specialists, this investigation provides a clinically rigorous estimate of PD prevalence in a high-risk cohort, in contrast to prior studies relying on self-reported data. These findings highlight the potential value of integrated urological and metabolic assessment in patients with DD. However, because our study is cross-sectional, causal inferences regarding the relationship between clinical or metabolic factors and PD cannot be made. Nevertheless, the benefit of systematic screening for PD in this population remains uncertain, and further longitudinal studies are needed to clarify patient benefit and guide clinical practice.
